# Mimicking the Kidney: A Key Role in Organ-on-Chip Development

**DOI:** 10.3390/mi7070126

**Published:** 2016-07-20

**Authors:** Roberto Paoli, Josep Samitier

**Affiliations:** 1 Nanobioengineering Laboratory, Institute for Bioengineering of Catalonia (IBEC), Barcelona 08028, Spain; rpaoli@ibecbarcelona.eu; 2 Centro de Investigación Biomédica en Red de Bioingeniería, Biomateriales y Nanomedicina (CIBER-BBN), Madrid 28029, Spain; 3 Department of Electronics, Universitat de Barcelona, Barcelona 08028, Spain

**Keywords:** organ-on-chip, kidney, nephron-on-chip, disease model, drug discovery

## Abstract

Pharmaceutical drug screening and research into diseases call for significant improvement in the effectiveness of current in vitro models. Better models would reduce the likelihood of costly failures at later drug development stages, while limiting or possibly even avoiding the use of animal models. In this regard, promising advances have recently been made by the so-called “organ-on-chip” (OOC) technology. By combining cell culture with microfluidics, biomedical researchers have started to develop microengineered models of the functional units of human organs. With the capacity to mimic physiological microenvironments and vascular perfusion, OOC devices allow the reproduction of tissue- and organ-level functions. When considering drug testing, nephrotoxicity is a major cause of attrition during pre-clinical, clinical, and post-approval stages. Renal toxicity accounts for 19% of total dropouts during phase III drug evaluation—more than half the drugs abandoned because of safety concerns. Mimicking the functional unit of the kidney, namely the nephron, is therefore a crucial objective. Here we provide an extensive review of the studies focused on the development of a nephron-on-chip device.

## 1. Introduction

In the last 60 years, most scientific and technological contributions to drug research and development (R&D) have brought about major advances; however, the pharmaceutical industry has been facing increasing production costs and a decrease in efficacy of R&D process [1,2]. The number of new drugs brought to market by the global bio-technology and pharmaceutical industries per billion US dollars of R&D investment has declined steadily in this period [1,3]. Although scientific and technological inputs into R&D have seen major advances, have not been reflected in drug R&D efficiency and thus there has been no increase in the probability of a small molecule successfully completing clinical trials [1]. These observations highlight the critical need for new testing approaches to achieve reliable predictions of drug efficacy and safety in humans, as well as to reduce the number of costly unsuccessful clinical trials.

Pre-clinical trials use two main tools, namely in vitro and animal models. The former consist of conventional two-dimensional (2D) and three-dimensional (3D) cell cultures. Two-dimensional cultures are a valuable tool in biomedical research, but they generally cannot reproduce tissue-specific function, may alter phenotype expression [4], and still fail to predict drug activity in vivo [5,6,7]. Recent decades have witnessed the development of microengineered 3D tissue models that mimic the physiological properties of native tissue samples, thus overcoming the major limitations posed by conventional cell-based assays for drug screening purposes. To date, cells of diverse embryonic origin have been cultured on microporous, nanofibrous, and hydrogel scaffolds—the latter overcoming some architectural and mechanical limitations of the first two [8]. Hydrogels are crosslinked networks with a high water content; they can be formed by natural molecules or synthetic polymers [9], and they induce cells to polarize and to interact with neighboring cells. They can be fabricated in customized shapes, and they are highly permeable to cell culture medium, nutrients, and waste products generated during the metabolic process [10].

In recent decades, organ decellularization has opened up new perspectives for tissue engineering by providing scaffolds that allow cells not only to retain their phenotypic properties but also to mature within the protein mesh provided. However, 3D scaffolds still have limitations. On the one hand, it is difficult to monitor cell position and to later harvest cells for functional or biochemical analysis. On the other hand, although 3D scaffolds provide important information about cell homeostasis (i.e., specific topography, appropriate stiffness, among others), they continue to lack the mechanical stimuli found in true cellular microenvironments (i.e., fluid shear stress, tension and compression). 

In order to overcome these limitations, engineers have worked alongside biologists, physicists and physicians to integrate 3D structures and microfluidics—a line of study that has given rise to the development of the first microfluidic OOC prototypes. Microfluidics is the science and technology of systems that process or manipulate small (10^−9^ to 10^−18^ liters) amounts of fluids using channels with dimensions of tens to hundreds of micrometers [11]. This way, OOCs exploit small size and the different behavior of liquids in microchannels, such as laminar flow [11].

## 2. Microfluidic Organs-on-Chip

Microfluidic OOCs are advanced platforms designed to mimic physiological structures and continuous flow conditions, thus allowing the culture of cells in a friendlier microenvironment.

The word “organ” in OOC may be misleading. The idea is not to synthesize the whole organ but to reproduce the minimal part able to exhibit tissue- and organ-level functionalities. On the other hand, the word “chip” refers to the main fabrication technique, namely photolithographic etching, borrowed from the electronic microchip manufacturing industry.

This technique enables the transfer of geometric micrometric patterns from a photomask to a light-sensitive chemical photoresist on a substrate. Depending on the positive (or negative) formulation of the resist, exposure selectively alters its polymerization, thus allowing the removal of exposed (or unexposed) zones.

Microfluidics makes use of so-called “soft-lithography”, a technique that allows the reproduction of patterns from silicon wafers to biocompatible “soft” elastomeric materials. This reproduction is commonly achieved by pouring a liquid polymer (poly-dimethylsiloxane, PDMS) into a rigid mold and allowing it to polymerize into an optically clear, rubber-like material [12].

The first simpler systems consisted of a single type of cell cultured in a single perfused chamber with the aim to mimic the functions of a given tissue. Later on, researchers focused on reproducing interfaces between distinct tissues to reproduce complex integrated organ-level responses [13]. In order to achieve this goal, more intricate designs were used to culture cell types in channels interconnected by porous membranes. Complete and detailed protocols for the fabrication of functional lung- and gut-on-chip devices have been published [14]. Following a pioneering study by Shuler and coworkers [15], the latest advances are addressing interconnection between organs. Various organ-type models have been combined, both embedding them in a single device [16,17] and by using interconnected bioreactors [18,19]. Khademhosseini and colleagues recently reported on the use of elastomeric free-form blood vessels to interconnect OOC systems [20].

These studies have paved the way toward in vitro models that mimic the function of the human organism as a whole. Although significant technical progress is being made towards this goal, the challenge continues to be not only the fabrication of functional lab-on-a-chip devices but also the proper scaling of tissues in terms of size and metabolic output. Takayama and Wykswo and their respective colleagues recently compared traditional allometric scaling against two different approaches based on histological section subdivision or the metabolic rate scaling ratio [21,22].

Wikswo and colleagues also pointed out the following major engineering challenges [23]: fluidic control of milli- and micro-liter volumes; analytical chemistry in micro- and nano-liter volumes; appropriate organ vascularization; blood surrogate development; and the modeling of coupled organs. However, given the sheer magnitude of funding invested by larger agencies, these challenges could be addressed in a timely manner [23]. As a potential improvement to current microfabrication techniques, the 3D printing of scaffolds, both alone and directly with cells, has also been examined [24,25].

## 3. The Kidney

### 3.1. The Role of Kidneys in Drug R&D

The kidneys serve as a natural blood filter, removing water-soluble waste from the cardiovascular system while reabsorbing useful substances (water, glucose, amino acids, etc.). Furthermore, they contribute to homeostasis by regulating electrolytes and blood pressure and by maintaining an acid–base balance, among other functions. Despite accounting for only 0.5% of body weight, the two kidneys of a resting adult receive about the 22% (1.1 L/min, 1584 L/day) of total cardiac output [26].

**Figure 1:**
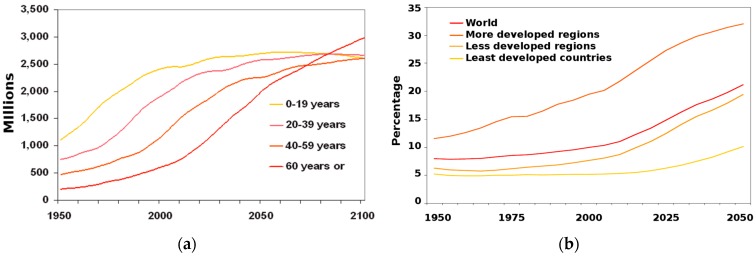
Trend in population ageing: (**a**) Population by broad age group: world, 1950–2100; (**b**) Proportion of the population aged 60 years or over: world and development regions 1950–2050. Reproduced from United Nations, *World Population Ageing*; 2013 [31], with permission of United Nations Publications.

Given that the kidneys play a major role in drug excretion [27], these organs take on particular relevance for drug R&D. Furthermore, nephrotoxicity, which damages kidneys, is a major cause of attrition during pre-clinical, clinical, and post-approval stages of pharmaceutical drug development [28]. Even after drug approval, nephrotoxicity resulting from exposure has been estimated to contribute to 19%–25% of all cases of Acute Kidney Injury (AKI) in critically ill patients [29]. Drugs cause roughly 20% of community- and hospital-acquired episodes of renal failure [30]. This figure reaches about 66% among the eldest population [30], thus placing an increasingly heavy burden on health care systems as the population ages (Figure 1) [31].

In light of the above considerations, the kidneys take on particular relevance in pharmacokinetics (PK) studies. The guidelines of both the Food and Drug Administration (FDA) and the European Medicines Agency (EMA) recommend that a PK study should be carried out during the development of a drug that is likely to be used in patients with impaired renal function [32,33]. In particular, they recommend characterizing drug PK in patients with different degrees of renal dysfunction and in the healthy population. This approach is needed to evaluate dosage adjustment; however, even when adjustments to recommended doses are followed, adverse reactions in patients with renal dysfunction are common [34]. Given this scenario, research into drug development and disease urgently need new tools that better mimic kidney function.

### 3.2. The Kidney Functional Unit: The Nephron

The specific structural and functional unit of the kidney is the nephron. Each kidney is formed by 800,000 to 1,200,000 nephrons [26,35], each of them located across the cortex and the medulla. 

Nephrons consist of a renal corpuscle connected to a complicated and twisted hollow tube composed of a single cell layer and consisting of three more sections, namely the proximal tubule, the intermediate tubule, and the distal tubule [26,35].

The renal corpuscle, also referred to as the glomerulus, is formed by glomerular capillaries and the Bowman capsule. The glomerulus is responsible for renal ultrafiltration—an ultra-selective filtering of blood plasma. Filtration is size-, charge- and shape- selective. This high selectivity occurs due to the complex structure of the glomerular barrier [36], the main components of which are the glomerulus epithelial cells, the podocytes, which have size-selective slit-shaped nanopores in their foot processes, and the glomerular endothelium, which has a charge-selective surface. In general, all molecules larger than 4.2 nm cannot cross the glomerular filtration barrier while those smaller than 2 nm can. Those falling in between these values are rejected or allowed to cross the barrier depending on their charge and shape. In particular, proteins (negatively charged) tend to be retained in the blood flow.

**Figure 2:**
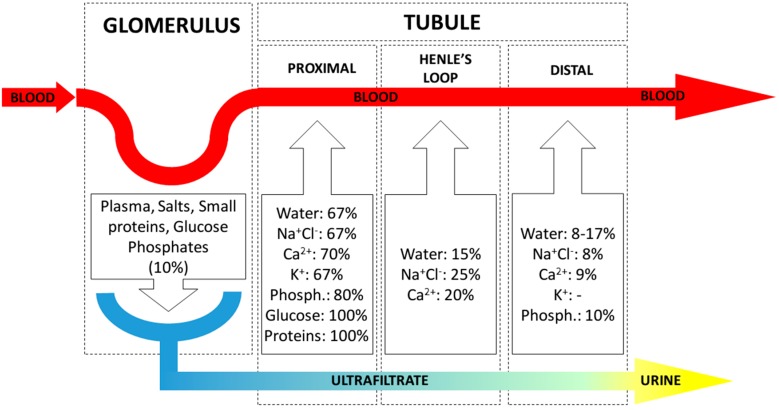
Renal ultrafiltration and reabsorption across the main sections of the nephron. Ultrafiltration from blood to ultrafiltrate takes place in the glomerulus. Reabsorption occurs across the tubule; in each section, reabsorption percentages of filtered amount are shown for the most relevant substances [26].

In contrast, the renal tubule reabsorbs water and pre-filtered useful substances toward the circulatory system. This process is mediated both by osmotic pressure, as well as by active transport by the tubular epithelial cells. Most reabsorption is performed in the proximal section of the tubule (Figure 2).

## 4. Kidney Disease Modeling

### 4.1. Major Kidney Diseases

Kidney conditions can usually be divided into two general subgroups depending on the extent of renal function impairment. AKI, previously called acute renal failure, is an abrupt loss of kidney function that usually develops within 48 h [37]. AKI can be reversed in some cases, but in others it is fatal. Chronic Kidney Disease (CKD), also known as chronic renal disease, includes any condition that causes reduced kidney function over a period of at least three months [38]. CKD is classified into five stages on the basis of the severity of renal impairment, stage 5 denoting End-stage Renal Disease (ESRD), meaning total and permanent kidney failure. Both AKI and CKD can arise from various conditions, either related to the kidneys, other organs, or systemic diseases. With respect to kidney-related diseases, some have attracted the attention of the research community due to lack of therapeutic options and knowledge of the underlying mechanisms involved. 

Glomerulonephritis (GN) is an inflammatory disease that compromises the glomeruli. One of the major features of this condition is ultrafiltration and renal function impairment. Although the causes vary (i.e., post-infectious GN, Immunoglobulin A nephropathy, anti-glomerular basement membrane antibody disease, ANCA-associated vasculitis and lupus nephritis), GN leads mainly to structural defects in podocytes, one of the cell types that ensures proper kidney function.

Polycystic Kidney Disease (PKD) is a group of monogenic disorders that result in renal cyst development [39]. Its most common variant, Autosomal Dominant Polycystic Kidney Disease (ADPKD), accounts for 10% of total ESRD diagnoses in the United States and Europe [40]. The cysts destroy the renal tubular epithelium, culminating in fibrosis, renal architectural disorganization and ultimately kidney failure. Thus far, the precise mechanisms leading to cyst formation and ADPKD progression have not been fully elucidated, and ADPKD treatment is largely supportive [41].

Renal Fibrosis is a direct consequence of the kidney’s limited capacity to regenerate after injury [42]. Fibrosis involves excess accumulation of extra-cellular matrix (ECM, mainly composed of collagen) and usually results in loss of function when normal tissue is replaced with scar tissue [43]. In spite of considerable advances in related research in 2015, renal fibrosis calls for greater research efforts [44].

Kidney Stones are a common urological problem, affecting approximately 10% of men and 6% of women and with an increasing prevalence over time in many developed countries [45,46]. Many aspects underlying of renal stone formation remain unclear; therefore, a better understanding of this intricate mechanism may pave the way to the development of a novel strategy to prevent this disease [47].

### 4.2. Current Models of Kidney Disease

The gold standard through which to study kidney diseases is by using patient clinical information [48]. While this method allows for exact reproduction of patient physiology, genetics, and environment, it has limitations, such as uncontrollable variation in genetic and environmental factors, availability and willingness of patients, and impossibility to perform genetic and biochemical experimentation [48]. Animal models are commonly used instead, providing a more controllable experimental system while maintaining the overall complexity of in vivo physiology. The main species used in kidney research are rats, mice, dogs and pigs [49]. Transgenic models have also been used to address genetic disorders [50] and the participation of cell lineages in physiological and pathological mechanisms [51].

Unlike animal models, 2D cell culture provides data in easily exploitable and genetically controlled environments. In the past, the main sources of cells, both primary and immortalized, were rats, dogs and humans. Recent efforts have moved towards 3D cell cultures, using novel scaffolds to grow cells. Cells or tubule pieces are most often embedded in hydrogel systems, where they form randomly spaced and aligned structures [48]. For example, cultured primary kidney epithelial cells have been isolated from ADPKD cysts to study the role of ouabain in the development of the disease [52]. By culturing kidney cells from ADPKD patients in a 3D collagen type I matrix, those authors discovered steroid hormone-induced cystic cell proliferation, a process that accelerates cyst formation.

Other studies involved the use of decellularized kidneys as 3D scaffolds. Techniques have been developed to decellularize rat [53,54,55], porcine [55,56,57], monkey [58,59,60], and human kidneys [55].

Many attempts have been made to mimic the complex structure and cell interaction of the glomerulus. Immortalized human podocytes and mesangial and glomerular endothelial cells have been established [61,62,63]; however, complete characterization of these cell lines in 2D culture has yet to be achieved [48]. Glomerular endothelial cells and podocytes have also been co-cultured on opposite sides of a nanofiber collagen type I/polycaprolactone scaffold [64].

Recent studies succeeded in generating kidney organoids via the directed differentiation of human pluripotent stem cells [65,66,67,68]. Organoids are 3D organ-buds grown in vitro that show realistic micro-anatomy. Their applications for the study of kidney physiology and pathology are promising. Recent research has addressed the nephrotoxicity of pharmaceutical agents [65,69], as well as the physiological growth and development of kidney-related structures [70]. 

### 4.3. Limitations of Current Models

While the models mentioned in the previous subsection address the lack of complex 3D tissue architecture and the interactions found in 2D cultures in vitro, they continue to have important limitations. Animal models vary significantly from humans in terms of gene expression. Moreover, there are significant ethical and economic issues concerning their use. Hydrogel-based models have the disadvantage of rapid degradation as a result of cellular remodeling and relatively low mechanical integrity, thus reducing their capacity to withstand the shear forces caused by fluid flow and limiting their utility for sustained cultivation [48]. Decellularized kidneys inherently lack the properties required for high-throughput studies. The glomerular tri-layered model is promising, though culture is performed in static, non-physiological conditions. With regards to organoids, the vascular environment in vitro is still far from resembling that of an adult organ [71].

## 5. Microfluidics Kidney-on-Chip

The first attempts to reproduce a kidney functional unit on a microfluidic device date back to 2001, when Essig and colleagues cultured a proximal tubule from C57Bl/6 mice and from the LLC-PK1 bovine cell line under flow conditions [72]. They discovered how tubular flow affects the phenotype of renal epithelial cells and proposed that flow-induced mechanical strains could be a determinant of tubule-interstitial lesions during the progression of renal diseases. The device used was itself simple, involving the assembly of two glass slides to form a parallel plate chamber (0.5 mm × 20 mm × 70 mm) and application of a laminar flow of 1 and 5 mL/min.

Later on, in 2007, Baoudin and colleagues developed a PDMS device with functional living cell microchambers interconnected by a microfluidic network that allowed continuous renal tubular cell feeding and waste removal control [73]. The PDMS device was formed by two layers, each with a depth of 100 μm. The first layer comprised a series of microchambers (300 μm × 300 μm) and microchannels (400 μm × 150 μm), while the second provided an inlet microchannel network and a chamber to distribute the culture medium. Those authors cultured Madin Darby Canine Kidney (MDCK) cells at various initial concentrations (2.5~5 × 10^5^ cells·cm^−2^) and flow rates (0, 10, 25, 50 µL·min^−1^). The results revealed the greatest proliferation during dynamic culture (10 µL·min^−1^). Basic chronic toxicity was assessed by adding ammonium chloride (5 and 10 mM) to the culture media, obtaining a considerable reduction in proliferation.

In 2008, Borenstein’s group developed a computational design of an ideal nephron-on-chip device composed by three steps, reproducing the glomerular, tubular and Henle’s loop functions, respectively [74]. The design used physiological dimensions for the channels in each step and computed flowrates were also coherent with physiological values [35]. Nonetheless, the study was limited to numerical simulations of urea reabsorption, and no further practical application of this design can be found in the literature.

**Figure 3:**
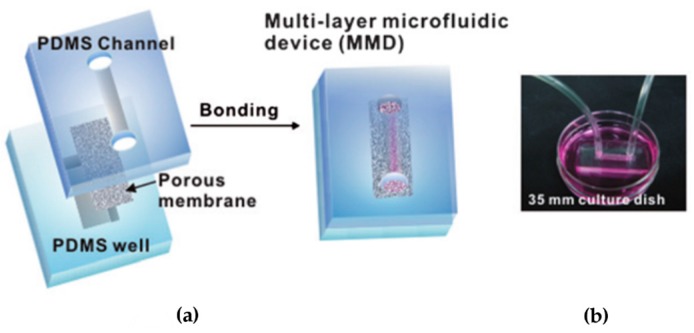
Fabrication and operation of a proximal tubule on a chip by Jang et al. [75]. (**a**) Device assembly, sandwiching together a PDMS channel, polyester membrane, and PDMS reservoir using plasma bonding; (**b**) Photograph showing the device, placed on a culture dish containing outer tubular fluid, connected to the fluidic setup. Reproduced from [75] with permission of the Royal Society of Chemistry. All rights reserved.

In 2010, another study addressed the effects of flow on renal cell culture [75]. The PDMS device included a porous membrane (Ø = 0.4 μm) for first time, which was used to separate a flow and a static compartment. The flow layer consisted of a single microfluidic channel (1 mm × 1 cm × 100 μm), while the lower layer was formed by a PDMS well (2 mm × 0.6 cm × 1~2 mm). The membrane was then sandwiched irreversibly between the two layers after oxygen plasma treatment (Figure 3).

Control devices were developed using the same PDMS channel bonded to a glass substrate. Both experimental and control devices were functionalized with fibronectin (10 μg·mL^−1^ for 1 h) to improve cell adhesion.

Primary rat inner medullary collecting duct (IMCD) cells were seeded and cultured statically in the device for three days up to confluency; later, they were exposed to a fluid shear stress of 0.1 Pa for 5 h while provided with nutrients from the static compartment containing outer tubular fluid. The results were compared with a control where no flow or basal/apical region was present. The authors observed conformational cellular polarization. IMCD cells recognized their apical and basolateral surfaces within the device. Fluid shear stress resulted in a three-fold increase in cell height and greater resistance to oxidative stress. Furthermore, molecular transport (water and Na uptake by Aquaporin 2) was found to be functional and responsive to hormone stimulation (Vasopressin, Aldosterone).

Another study worthy of mention, although with a different application, is Roy’s and Fissell’s artificial kidney. Roy and Fissell designed a device to mimic renal ultrafiltration for implantable hemodialysis purposes [76]. They used silicon nanoporous membranes with a geometrically controlled pore size to reproduce ultrafiltration [77] and proximal tubule cells to improve reabsorption. The device showed promising results with respect to an implantable artificial kidney [78] and has recently been tested in dogs [79].

**Figure 4:**
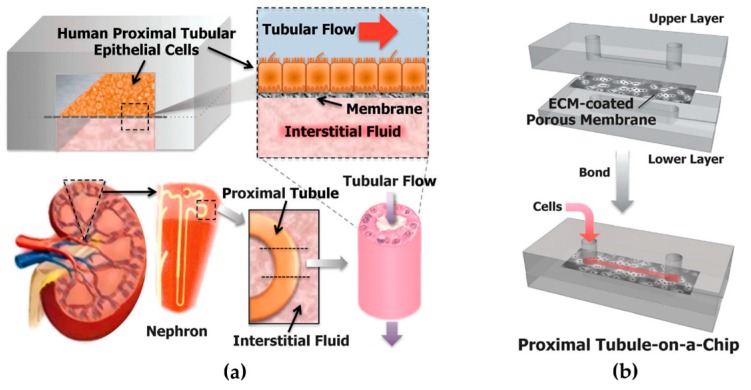
Design of the human kidney proximal tubule-on-a-chip by Ingber’s group [6]. (**a**) The microfluidic device consists of two PDMS channels, resembling the proximal tubule and interstitial space, separated by an ECM-coated porous membrane. HPTECs are cultured on top of the membrane, in the presence of a physiological level of apical fluid shear stress. (**b**) Device assembly: The upper layer, polyester porous membrane, and lower layer are bonded together through surface plasma treatment. Reproduced from [6] with permission of the Royal Society of Chemistry. All rights reserved.

The first approach to develop a human proximal tubule-on-a-chip was published by Ingber’s group in 2013 [6]. The device was a slightly modified version of [75], where the dimensions of the lower PDMS well were modified (1.1 mm × 1.1 cm × 3 mm) while the microfluidic channel and the membrane remained the same (Figure 4). Primary Human Proximal Tubule Epithelial Cells (HPTECs) were plated on top of the membrane (2 × 10^5^ cells·cm^2^) under static conditions and cultured under a moderate flow shear stress (0.02 Pa). Control cells were plated on similar ECM-coated porous polyester membranes but cultured in the absence of flow in traditional Transwell inserts. After three days, the cells subjected to physiological tubular flow showed enhanced differentiation, as revealed by increased primary cilium formation, alkaline phosphatase activity, albumin transport, and glucose reabsorption.

Furthermore, the device was tested for drug-induced (cisplatin) toxicity response. A more specific toxicity response was found in a “proximal tubule-on-a-chip” than in traditional culture, as cells were healthier at the baseline. Moreover, a higher protective response was observed while co-administering OCT-2 inhibitor, as well as a greater recover rate after acute cisplatin damage.

Some researchers have attempted to model specific renal pathologic conditions, like renal fibrosis [80] and kidney stone formation [81]. These studies do not seek to reproduce the whole organ functional unit but rather specific parts or a specific disease condition.

Moll and colleagues addressed the role of HK-2 cells in renal fibrosis. They cultured renal cells on top of human dermal fibroblasts in order to examine how interactions between the two cell types contribute to cisplatin-induced kidney injury. Tubular cells were found to affect fibroblast gene expression.

**Figure 5:**
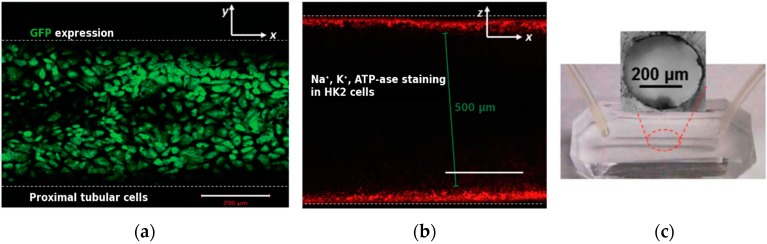
Kidney stone formation, from Wei and colleagues [81]. (**a**) Green fluorescence protein (GFP) expression showing typical basolateral staining in the monolayer of cells on the microchannel wall; (**b**) Channel cross-section showing the distribution of key plasma membrane protein Na^+^/K^+^-ATPase. Scale bars are 200 μm; (**c**) Photograph of the device with circular cross-section. Reproduced from [81] with permission of the Royal Society of Chemistry. All rights reserved.

Wei and colleagues studied kidney stone formation within a PDMS device with a 400-μm cylindrical microfluidic channel (Figure 5) [81]. The device was functionalized with fibronectin and seeded with proximal tubule epithelial cells from the immortalized HK-2 line. After injection of CaCl_2_ and Na_3_PO_4_, in situ formation of calcium phosphate stones was observed in real time using Raman spectroscopy.

**Figure 6:**
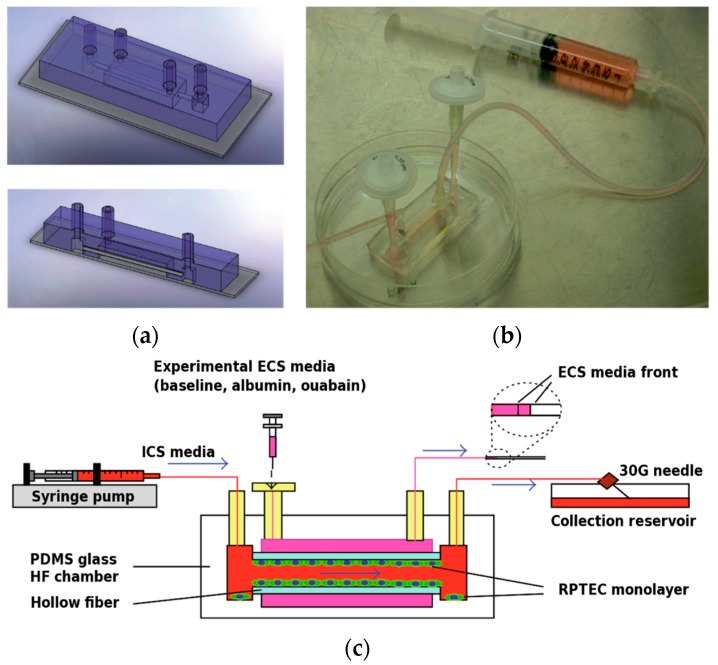
A Fibrin-Based Tissue-Engineered Renal Proximal Tubule for Bioartificial Kidney Devices: Development, Characterization and In Vitro Transport Study (2013). (**a**) Schematic and (**b**) image of the “lab-on-a-chip” hollow-fiber bioreactor. (**c**) Set-up for perfusion studies. Reprinted from [82] under CC-BY 3.0 with permission of Hindawi Publishing Corporation.

Another different technical approach for kidney-on-chip development used embedding dialysis-like hollow fiber membranes inside a PDMS device [82]. The hollow fiber (outer diameter: 780 μm, inner diameter: 490 μm) was assembled together with the PDMS apical and basal chamber on a microscope slide (Figure 6). The membrane was sterilized with ethanol, incubated with 5 mg/mL bovine fibrinogen and 50 U/mL bovine thrombin and seeded four times with HPTEC in order to cover the whole membrane surface. Culture was then performed under flow condition for seven days. Successive fluorescent immunostaining and electron microscopy revealed a confluent HPTEC monolayer with polarized expression of proximal tubule and ion transport markers, as well as the formation of microvilli on the apical side. Tubular reabsorption was tested as baseline and with albumin and Na^+^/K^+^-ATPase inhibitor, and the results suggested that reabsorption is mediated by active transport.

In 2013, a group at Huazhong University in China also developed a microfluidic device that was an interface point between dialyzer miniaturization and an OOC device [83]. Microfluidic titanium layers with a serpentine channel (1 mm wide, 500 μm height) were assembled together, separated by porous dialysis membranes. Various membrane materials (Polyethersulfone—PES, Mixed Cellulose Ester—MCE, Regenerated Cellulose—RC) were tested. The device was modular, meaning that layers could be added to increase the total interchange surface.

Two types of immortalized cells, namely HK-2 and HUVEC, were cultured in different layers. Cell proliferation was tested for incubation in static and dynamic conditions (adding a 2-h dialysis after the first 24 h of incubation). Filtration capacity was tested by measuring urea nitrogen and vitamin B12 clearance after single-pass dialysis. Sodium reabsorption was tested by irrigating both the blood and dialysate channel with perfusion solution containing 140 mmol/L Na^+^.

The authors of that study reported a two-fold improvement in proliferation when cells were exposed to flow stimulation. The best results were obtained with PES and MCE membranes. Urea nitrogen and B12 clearance with a PES membrane reached physiological values when using a 6-layer chip. Sodium reabsorption mediated by HK-2 cells was demonstrated by comparing physiologic results with negative controls in which active cell transport was inhibited by addition of ouabain. The 6-layer chip also reached physiological ammoniagenesis values, which are essential for the kidney to maintain acid-base homeostasis in the body. 

**Figure 7:**
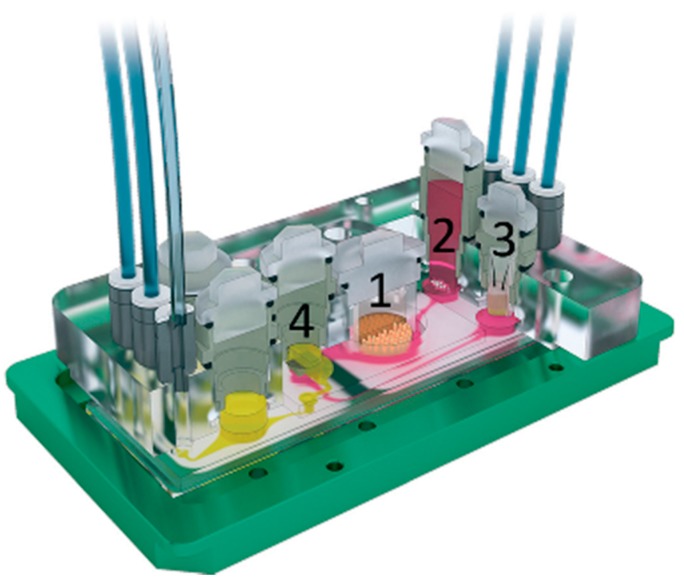
3D view of microfluidic four-organ-chip device by Maschmeyer et al. [7], (footprint: 76 mm × 25 mm; height: 3 mm). Intestine (1), liver (2), skin (3), and kidney (4) tissue culture compartments are interconnected by a surrogate blood flow circuit (pink) and an excretory flow circuit (yellow). Reproduced from [7] with permission of the Royal Society of Chemistry. All rights reserved.

A recent study moved one step forward by performing a toxicology analysis multi-organ-on-chip device [7]. Equipped with a standard microscope slide footprint, the device consisted of four interconnected compartments to culture intestine, liver, skin and kidney tissues (respectively, 1, 2, 3 and 4 in Figure 7). The chambers were interconnected by a microfluidic channel for surrogate blood flow which overlapped in the tubular region with a second microfluidic circuit that allowed drainage of fluid excreted by renal epithelial cells. Each microfluidic circuit was actuated by a dedicated on-chip peristaltic micropump. Intestine and liver cells were from a primary source, while skin and kidney ones were from immortalized lines. Tubular cells showed correct polarization of Na^+^/K^+^-ATPase. Furthermore, together with small intestine cells, they were able to maintain a stable glucose gradient balance between the intestinal lumen, the surrogate blood circuit, and the excretory circuit of the system. 

## 6. Conclusions

Microfluidics has brought about significant advances in the development of in vitro micro-physiological systems able to reproduce in vivo organ function. These new tools pave the way for drug development and clinical research into disease and may avoid the need for animal models in the future while producing more information. Of all the organs that researchers have attempted to reproduce, the kidney heads the field because of its critical relevance in drug development. The kidneys are pivotal in most drug removal processes, as well as in drug safety evaluation.

The field of microfluidics has come a long way since the first effort to reproduce the kidney functional unit back in 2001, and considerable breakthroughs have been made toward a fully functional OOC platform. However, there are still challenges and limitations to overcome.

Most studies focus exclusively on the tubular section of the nephron; however, there is a wide range of illnesses or renal dysfunctions that are related to impaired glomerulus activity. In particularly, the alteration of podocyte foot processes is responsible for inefficient blood ultrafiltration, leading to proteinuria and other pathological conditions. A great challenge would be the addition of a glomerular barrier to a nephron-on-chip, possibly using podocytes. In this regard, several issues need to be addressed as podocytes culture is complicated [84] and requires precise shear stress control [85].

Another limitation is that only a few studies have worked with primary human cells. While immortalized cell lines are easier to culture in vitro, they may introduce their slight phenotypic mismatches with in vivo tissues and they are not patient-specific [86].

A general approach for developing an OOC is to identify key aspects of the geometrical, mechanical and bio-chemical microenvironment of the tissue of interest. In this regard, little attention has been devoted to the geometrical aspect. For instance, in many developments, channel dimensions are much larger than those of the physiological tubule. 

A further pending improvement, which continues to be a challenge in OOC technology, is the inclusion of sensors. Many analyses are still performed outside the device, using standard methods. To obtain a truly efficient and cost-effective device, it would be pertinent to equip the device with sensing capacity and thereby obtain direct data. Although studies have been published on other organs and tissues, each one requires a specifically designed device. It would be opportune to develop modular systems or even designs to include various readouts in function of the parameters required by each experiment. 

The paradox of achieving “perfect” models was put into perspective by Borges, highlighting the uselessness of an empire map the size of an empire [87]. However, there is still scope to increase the complexity of current models while conserving their utility. Nephron-on-chip technology has experienced significant development over the last decade and promises to pave the way for novel tools to study the effects of pharmaceutical agents and disease models in an organ of particular relevance like the kidney.

## Abbreviations

The following abbreviations are used in this manuscript:

2D: Two-dimensional
3D: Three-dimensional
AKI: Acute Kidney Injury
(AD)PKD: (Autosomal Dominant) Polycystic Kidney Disease
ANCA: Anti-neutrophil cytoplasmic antibodies
CKD: Chronic Kidney Disease
ECM: Extra-cellular matrix
EMA: European Medicines Agency
ESRD: End-Stage Renal Disease
FDA: Food and Drug Administration
GN: Glomerulonephritis
HPTEC: Human Proximal Tubular Epithelial Cells
HUVEC: Human Umbilical Vein Endothelial Cells
ID: Inner diameter
IMCD: Inner Medullary Collecting Duct
MCE: Mixed Cellulose Ester
MDCK: Madin Darby Canine Kidney
OD: Outer Diameter
OOC: Organ-on-chip
PES: Polyethersulfone
PK: Pharmacokinetics
PDMS: Poly-dimethylsiloxane
R&D: Drug research and development
RC: Regenerated cellulose
